# Effort Perception is Made More Accurate with More Effort and When Cooperating with Slackers

**DOI:** 10.1038/s41598-019-53646-9

**Published:** 2019-11-25

**Authors:** Paul Ibbotson, Christoph Hauert, Richard Walker

**Affiliations:** 1 School of Education, Childhood Youth and Sport, Open University, Walton Hall, Milton Keynes MK7 6AA England; 2 Department of Mathematics, The University of British Columbia, 1984 Mathematics Road, Vancouver, B.C. V6T 1Z2 Canada; 3 School of Computing and Communications, Open University, Walton Hall, Milton Keynes MK7 6AA England

**Keywords:** Evolutionary theory, Human behaviour

## Abstract

Recent research on the conditions that facilitate cooperation is limited by a factor that has yet to be established: the *accuracy* of effort perception. Accuracy matters because the fitness of cooperative strategies depends not just on being able to perceive others’ effort but to perceive their true effort. In an experiment using a novel effort-tracker methodology, we calculate the accuracy of human effort perceptions and show that accuracy is boosted by more absolute effort (regardless of relative effort) and when cooperating with a “slacker” rather than an “altruist”. A formal model shows how such an effort-prober strategy is likely to be an adaptive solution because it gives would-be collaborators information on when to abort ventures that are not in their interest and opt for ones that are. This serves as a precautionary measure against systematic exploitation by extortionist strategies and a descent into uncooperativeness. As such, it is likely that humans have a bias to minimize mistakes in effort perception that would commit them to a disadvantageous effort-reward relationship. Overall we find support for the idea that humans have evolved smart effort detection systems that are made more accurate by those contexts most relevant for cooperative tasks.

## Introduction

The degree to which humans cooperate with each other is unique in the natural world. It is woven in to the cultural fabric of our lives and without it many of our most human achievements – law, government, trade, education, language – would not be possible^1–8^. Cooperation has attracted a lot of attention not just because humans are an ultra-cooperative species but that it appears to run contrary to the fundamentally competitive nature of evolution by natural selection^9^.

Cooperation can actually enable individuals to become better competitors, because the cost of short-term unselfish behavior is repaid by the selfish long-run benefits^8–10^. Although cooperation can yield rewards it remains a fundamentally risky business because failed cooperation can leave an individual worse off than no cooperation at all^11^. For this reason people are choosy about whom they cooperate with^12,13^ and mitigate some of the risk by collaborating with those with a reputation for fairness^14–16^ or by shunning or punishing those who free-ride on others^17–20^.

The costs and benefits of cooperation have been most frequently quantified using monetary payoffs or penalties. However, for the majority of species and for most of human history the costs of collaboration are better characterized as time and effort. Much of human cooperation is fundamentally mutualistic in nature (foraging, hunting, fishing), so the effort of each cooperative partner must be monitored and coordinated and in order that *anybody* gains the rewards of a collaborative venture^11,21^. Recently, attention has turned to the circumstances under which people are willing to invest effort. For example, it has recently been shown that people are likely to persist longer, are more committed and perform better in a cooperative task when they perceive their partner to have invested more effort^22,23^. While perceptions are important, the significance of these findings is limited by a underlying factor that has not been investigated: the *accuracy* of effort perception. Accuracy matters because the fitness of behavioral strategies depends not just on being able to perceive others’ effort but to be perceive their true effort. For example, cooperators whose perceptions of effort only loosely correspond with reality are likely to be out-competed by those who can systematically avoid their fair share of effort but can still gain the rewards of cooperation. Therefore one would expect pressure to evolve ever-more accurate effort detection cognition, heightened by those contexts most relevant for cooperative tasks^24^. We test this hypothesis by systematically varying relative effort and asking people to judge the effort of others in an online cooperative game. In this game, two players invest effort by pushing virtual balls up a ramp and into a bucket controlled by clicks of the mouse. The rewards are shared 50/50 between the players. As such, the game emphasizes the mutual interdependence of partners in the form of ‘sharing of the spoils’ – a behavior that seems to have played an important role in cementing cooperative exchanges in our evolutionary past^7,25,26^. The required response from participants was simple: who put in more effort into the task, you or your partner? (see Methods).

To determine the validity of relative effort perception, we needed to systematically vary the effort between two collaborators. We could not and did not want to control the absolute effort of the human participants and for that reason, the ‘other player’ was in fact controlled by a computer algorithm designed to vary the effort as a ratio of the participant’s effort (Supplementary Information). By doing so we could measure each participants’ absolute effort, their effort relative to their virtual collaborator, and objectively determine the accuracy of perceived effort.

## Result

**Figure 1:**
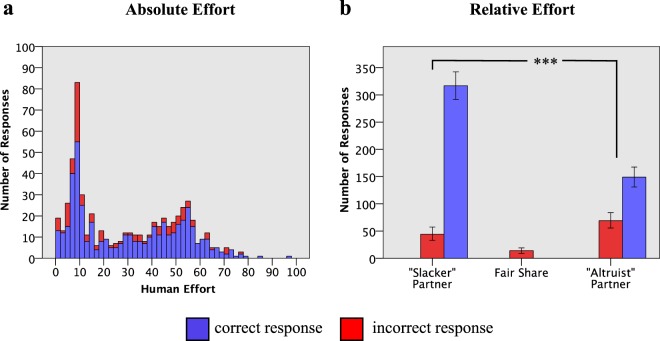
Absolute effort (**a**) shows a histogram of the number of responses (y-axis) of participants in the experiment providing x amount of effort (x-axis is divided into bin sizes of 2). For each amount of participant effort their perception about the computer’s effort, could either be right or wrong, and so the bar is divided into correct responses and incorrect responses stacked on top. Relative effort (**b**) is calculated as a ratio of the human effort to the computer player, where “slacker” indicates that the computer player was putting in less effort than the human, “fair share” that they were matched in effort and “altruist” where the computer was putting in more effort. Error bars represent 95% CIs.

Participants correctly predicted whether their partner was an ‘altruist’ or a ‘slacker’ with an accuracy of 78.6% (Fig. 1a). A Chi-squared test revealed a significant difference between Altruist and Slacker conditions in the number of correct and incorrect responses χ^2^ (1) = 32.78, *p* < 0.001 (Fig. 1b), such that when human participants were paired with a slacker, they were on average more accurate in their perception of effort.

**Figure 2:**
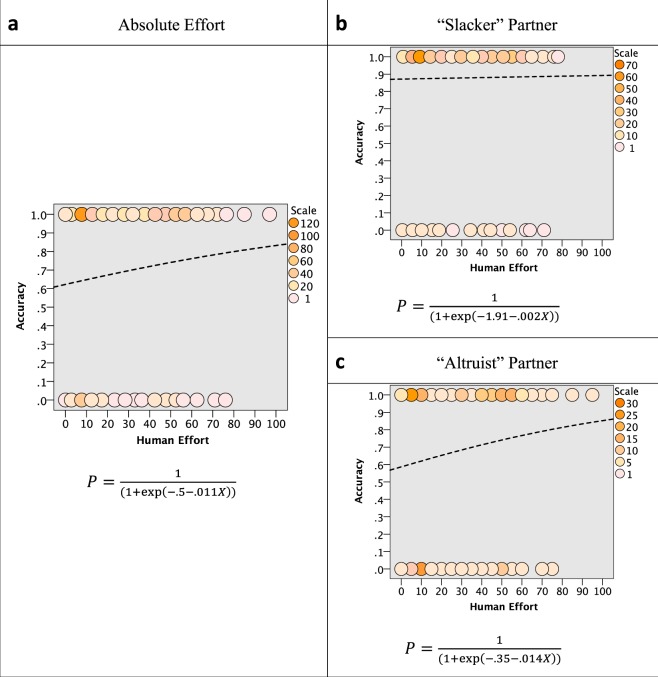
Predicted probabilities of participants giving an accurate response based on binary logistic regression. The intensity of the circles denote the frequency of participants response for a given effort, see individual scales. Note Fair Share was not tested as all 14 cases were incorrect responses.

To calculate the predicted probabilities of participants giving an accurate response across a continuous range we plotted a logistic curve of the general form $P=\frac{1}{(1+\exp (-{\rm{a}}-{\rm{bX}}))}$ where *P* is the predicted odds for accuracy (0–1), and *a* and *b* are parameters of the model fitted to the data. The specific values of these parameters are shown in Fig. 2 for Absolute Effort (a), as well as Slacker (b) and Altruist (c) partners. To investigate the hypothesis that *absolute* effort predicts accuracy of effort perception we conducted a binary logistic regression with accuracy (correct = 1, incorrect = 0) as our dependent variable and effort (number of clicks) as a predictor. The result was significant χ^2^ (1) = 5.708, *p* = 0.017, such that every unit increase in effort increased the logs odd for accuracy by 0.011, *p* = 0.019, 95% CIs [1.002, 1.021] (See Fig. 2a). This means that, regardless of the relative effort of their collaborator, the more effort people put into the task the more likely they were to make correct judgements about their partner’s effort, getting 78.6% of them correct, on average. We then spilt the absolute effort data by Slacker and Altruist conditions. Participants in the Slacker scored 87.8% correct and the model was not significant χ^2^ (1) = 0.079, *p* = 0.779, showing absolute effort does not predict accuracy in the slacker condition essentially because participants are already getting 87.8% correct (see Fig. 1b). That fact that it was not significant in this case does not allow us to talk about a meaningful change in the outcome variable from the predictor variable. Participants in the Altruist condition scored 68.3% correct and the model was significant χ^2^ (1) = 4.608, *p* = 0.035, such that for every unit increase in effort there was a predicted change in the logs odd for accuracy of 0.014, *p* = 0.019, 95% CIs [1.001, 1.028].

## Discussion

We investigated the accuracy of peoples’ perception of effort. Overall, people were largely accurate in their perceptions of others’ effort, correctly judging who is putting in more or less effort 78.6% of the time (Fig. 1a). However, there were certain contexts in which this accuracy was heightened. Effort perception was made more accurate when that partner was a “slacker” rather than an “altruist” (Fig. 1b). Effort perception of a partner was also made more accurate when people put in more effort themselves, regardless of what their partner was doing (Fig. 2a). In what follows we interpret our findings under the framework of Error Management Theory (EMT)^27,28^ which has provided evidence across a range of behaviors that these types of perceptual biases represent adaptive solutions to the decision-making problems of our evolutionary past^24,27,29–36^.

In everyday encounters, as in this experiment, people are required to make decisions about their potential collaborators under uncertainty and the judgments are therefore prone to error. A participant could believe their partner was putting in more effort when they are putting in less (false positive) or putting in less effort when they are putting in more (false negative). Whenever the payoff is not equal between false positives and negatives, it is likely that natural selection will favor cognitive biases that minimize whichever mistakes incur the greatest costs, rather than minimizing the total number of mistakes itself^29,37^. The consequences for wrongly attributing more effort to a partner, risks wasting effort on a cooperative venture that yields little rewards. The consequences for wrongly attributing less effort to a partner, risks abandoning a cooperative venture that could yield high (shared) rewards.

Risk and stakes increase with absolute effort because the more effort someone invests in a task the more they stand to gain but also the more they stand to lose: if a (big enough) reward does not materialize to offset the investment, they could be left in metabolic debt (starving) or bankrupt (dead). Thus, it makes sense that people get increasingly vigilant and acutely monitor the effort of others the more effort they commit themselves to the task. Relative effort perception is important to correctly perceive the partner’s effort in order to determine whether the costs-benefits of cooperation are not in an individual’s favor, and to reduce the chance to collaborate with a slacker, regardless of the absolute efforts of their partner.

Because we have established that perceptions of effort have a tight relationship to true effort, in both absolute and relative terms, this grounds behavioral strategies based on effort perception in the reality of energy expenditure. This opens the door for effort-strategies to form part of the human cognitive repertoire for keeping cooperation on track. For example, if it appears as if the distribution of effort amongst partners is such that no one will receive the reward, or simply that the goals of the partners are not aligned, it may be better to cut losses and switch to a strategy that secures a lower payoff, but with lower risks. Sensitivity to relative effort could therefore mitigate some of the risk of choosing cooperation because it provides information on whether and when to abort cooperative ventures that are not in their interest. There are abundant examples of withdrawal of cooperation in the face of a lack of effort and the ostracism of the slackers in the ethnographic record^38–41^. Recent modelling work shows probabilistic abstention in certain cooperative contexts can be an optimal strategy^42^. To investigate the advantage that an effort-probing strategy has on cooperation, we used the classic Stag-Hunt game^11,21^. In this scenario, two players can hunt their own low-value prey (hare) or they can coordinate their effort to catch the higher value prey (stag). The different outcomes are summarized with the payoff matrix (Table 1) where each partner’s share of the stag is normalized to one and the value of a hare is 0 < *a* < 1.

**Table 1:**
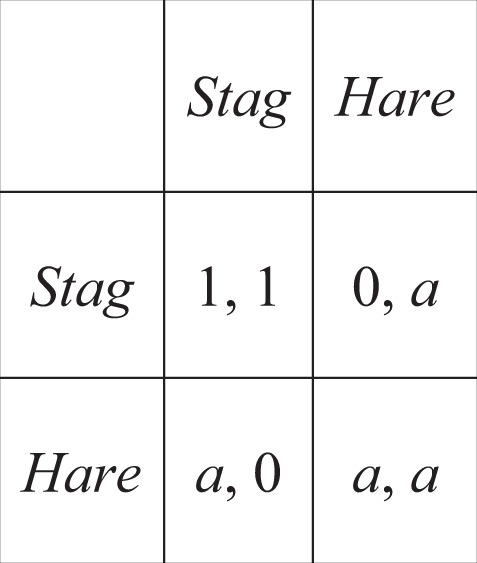
Stag-Hunt payoff matrix.

Hunting the stag is the efficient and mutually preferred outcome but for *a *> 1/2 catching a hare is the risk dominant strategy. This generates a conflict because each partner is aware that they need each other to capture the stag but hunting it alone means forsaking both options. Formally, introducing a *prober* strategy is capable of mitigating the risk of opting for *stag*. *Probers* make a small initial investment, *ε*≪1, (or series of small investments) and if the partner reciprocates they choose *stag* but if the partner fails to reciprocate they minimizes their losses by opting for *hare*. This results in an extended payoff matrix (Table 2) and introduces another coordination game between the *hare* and *prober* strategies, while *prober* and *stag* are neutral.

**Table 2:**
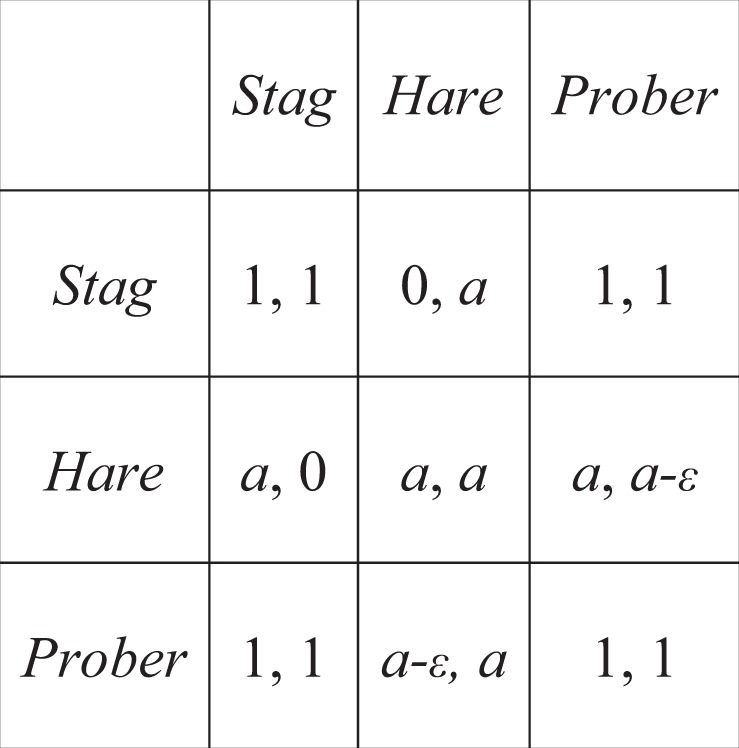
Extended Stag-Hunt Payoff Matrix.

In an evolutionary setting the dynamics of a population can be investigated based on the replicator equation, $\dot{x}={x}_{i}({f}_{i}-\bar{f})$ where ${x}_{i}$ denotes the frequencies of strategy $i$ with $\sum _{i}{x}_{i}=1$, ${f}_{i}$ their average payoff and $\bar{f}$ the average population payoff^43^. For stag-hunt interactions (see Eq. 1) this represents the classical case of a coordination game with bi-stable dynamics: both homogeneous population states are stable with everyone hunting (*x*_stag_ = 1) or (*x*_hare_ = 1), respectively^11^. Most importantly, however, once a population is trapped in the hare equilibrium it is very difficult to re-establish the mutually preferred cooperative stag hunts because of the associated risk and reliance on the partner’s behavior.

**Figure 3:**
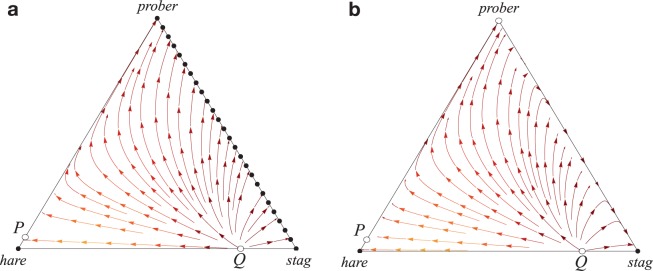
Simplex plot of replicator dynamics with three strategies stag, hare and prober. Each corner marks homogeneous populations of only the corresponding strategy. (**a**) With the help of prober essentially all population configurations eventually converge to everyone opting for stag or prober or any mixture of the two (the stag-prober-edge is neutral). Moreover, the threshold for prober to escape the homogeneous hare equilibrium is very small. In contrast, in the absence of prober, hare is risk dominant as reflected in the much larger basin of attraction of hare as compared to stag (bottom edge of the simplex). (**b**) The role of prober does not change if the costs ε apply regardless of the type of partner but now stag outperforms prober in the absence of hare such that stag becomes the sole, risk dominant strategy. Parameters: a = 0.8, ε = 0.01, i.e. probing costs are 1% of the benefit for successful coordination on the efficient outcome. In both scenarios the two unstable equilibria are located at Q = (1-a, a, 0) and P = (1-ε/(1-a), 0, ε/(1-a)).

The *prober* strategy provides an escape hatch out of the inefficient hare equilibrium and paves the way for successful coordination on hunting stags even under adverse conditions where *hare* is the risk dominant strategy in the absence of *prober* (Fig. 3a). With this change, *prober* and *stag* (or any mixture of the two) become the risk dominant strategy regardless of *a*.

Naturally, *prober* is a more sophisticated strategy than both unconditional *stag* or *hare* because it requires cognitive abilities to perceive the efforts of partners, compare it to its own and change behavior in response. Maintaining these cognitive abilities can be costly and would reduce the payoff of *prober* in payoff matrix (2) by some amount against any partner. However, this does not change the role of *prober* as a potent promoter for efficient coordination (Fig. 3b). In summary, this strategy efficiently reduces the risk of mutualistic endeavors because readily detecting shirking partners prevents wasted efforts and allows to abort cooperative ventures that are not in an individual’s interest. Introducing behavioral strategies that probe a partner’s effort at small costs effectively promotes cooperation by readily abandoning interactions with bleak perspectives. Moreover, the cognitive bias provides further protection against subtle exploitation through extortionist strategies^44^.

A strategy to categorize would-be collaborators entirely on the basis of their effort contribution cannot explain experimental evidence that shows adults and children are sensitive to difference between unwilling partners (e.g., exploitative free-riders) versus unable partners (e.g., unintentional uncooperativeness due to accident, illness or bad luck) even when effort is held constant^45,46^. However, acknowledging human sensitivity to the ‘unable-unwilling’ distinction does not rule out the role that relative effort perception plays in supporting cooperation when intentions, competence and luck are held constant and effort varies (a condition that contrasts with^46^). This sensitivity may be especially important when the outcomes of collaborative efforts are tightly tied to the relative efforts of the participants, the role of luck and mistakes is diminished and thus the need for psychological mechanisms to detect the difference between unwilling versus unable is also diminished. For example, some collaborative activities are by their nature lower-variance, lower risk ventures (e.g., foraging) when compared with others (e.g., hunting). In societies with more predictable diets, the effort one puts in is more proportionate to output one receives. Such economies predict a relatively increased role for relative effort sensitivity and the behavioral strategies (such as ‘Prober’) that are contingent upon it^38,47,48^.

In our Methodology, after balls fell off the edge of the ramp they were occluded behind the bucket. The total number of balls in the bucket was never revealed to participants to prevent them counting the relative frequency of balls in the bucket after the trial has finished. The most salient information they had available to them during the trial was the relative frequency that the balls were being pushed up the ramp – our measure of effort. Thus in our task outcome is directly proportionate to effort. Future variations on this methodology may want to develop a more indirect relationship between outcome and effort, such as a ball that slides back down the ramp if it has not been pushed within a certain time frame. Pilot experiments revealed that participants were sensitive to subtle social framing of the effort detection task, for example, instructing participants that the rewards will divided according to individual effort rather than 50/50 as in the present study. The fact that these sensitivities occurred with the exact same perceptual stimuli (balls moving up ramps) suggests that participants’ top-down knowledge of the cooperative task interacted with the bottom-up perception of effort.

Overall, we find support for the idea that humans have evolved accurate effort detection systems, heightened by those contexts most relevant for cooperative tasks^24^. Accuracy of effort, and not just perception of effort^22,23^, matters because increased metabolic expenditure in cooperative tasks is regulated by the true nature of the effort not by perceived effort.

Collaborative effort is needed for a wide range of cooperative activities from the more psychological (e.g., group problem solving^49^) to the more physical (e.g., cooperative pulling paradigm^25^). To the extent that all collaborative ventures pose the same basic problem of coordination they should engage similar mechanisms designed to mitigate the risk of disadvantageous partnerships. Thus we expect that the same heightened awareness of relative effort and absolute effort would be engaged across a range of physical and mental cooperative tasks as long as there is some public production of a good that is shared.

The modeling of an effort-prober strategy shows this is likely to be adaptive because it gives would-be collaborators information on when to abort ventures that are not in their interest and opt for ones that are. As such, it is likely that humans have a bias to minimize mistakes in effort perception that would commit them to a disadvantageous effort-reward ratio. This turns cooperation in to a less risky strategy than if no such bias existed. In conclusion, providing behavioral escape routes to abort social interactions^50^ may be one paradoxical way to boost cooperation because it serves as an insurance against incompatible goals and prevents exploitation through free-riders.

## Method

An online computer game was played by 190 undergraduates enrolled on a Psychology Module at a University in the UK.

All experimental protocols were approved by The Open University Research and Ethics Committee. All methods were performed in accordance with the relevant guidelines and regulations, including obtaining informed consent from all participants and their right to withdraw from the experiment and their right to withdraw their data. Each “game” consisted of 5 trials such that there were 947 data points (with 3 points of omission due to one participant not completing all 5 trials). Before the game began, they received the following instructions:

You are going to a play a series of games. Each game lasts 10 seconds. The aim of the game is to move balls into a bucket by pushing them up a ramp. Another player will be doing the same thing as you. You control your pushes by clicking the mouse anywhere inside the grey rectangle. The more effort you put in the more balls will go in the bucket. The other player controls their pushes by doing the same thing as you. At the end of the game the total number of balls in the bucket will be split 50/50 between you and the other player.

**Figure 4:**
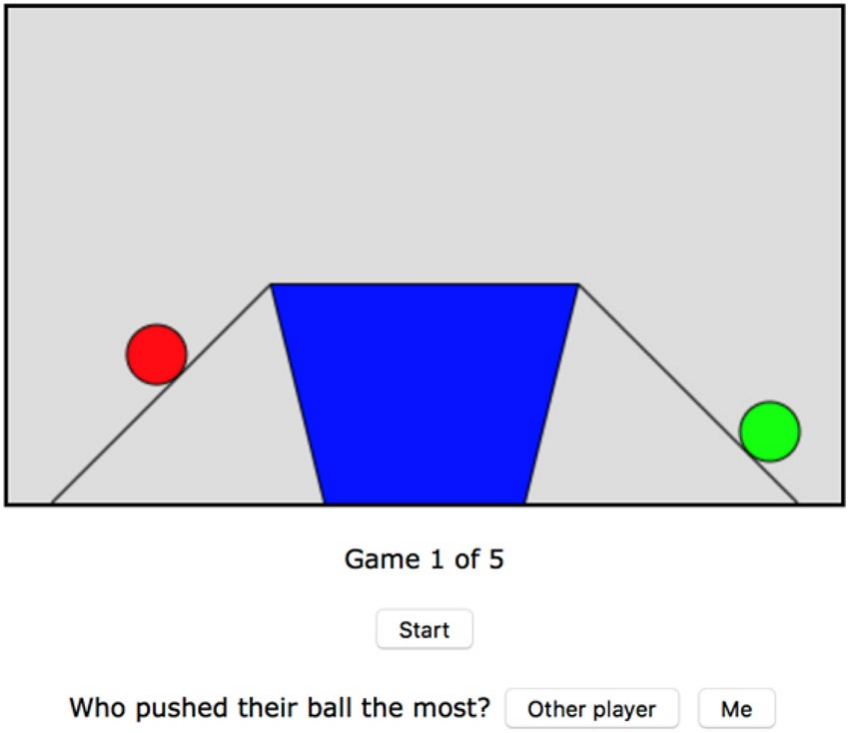
Example Screenshot of the Game. The left-right order of the ‘Other player’ and ‘Me’ buttons was randomized.

After participants had read these instructions, they played the game, and at the end of each game the participant’s task was simple: decide whether they put in more or less effort than the other player (Fig. 4).

Participants were not explicitly told that they would have the same partner over all 5 rounds but neither were they told the other player would change. We suspect the use of “player” and “*the* other player” rather than “players” lead most people to assume they were playing with one person over the 5 rounds.

## Supplementary information

Supplementary Info

## Data Availability

Source data available on request.
