# Supplementary material for: Effort Perception is Made More Accurate with More Effort and When Cooperating with Slackers
Source: Sci Rep. 2019 Nov 25;9:17491. doi: 10.1038/s41598-019-53646-9 (PMC6877554; doi:10.1038/s41598-019-53646-9)
Supplement: Supplementary file 1 — Supplementary Info [file 41598_2019_53646_MOESM1_ESM.pdf]

Effort Perception is Made More Accurate with More Effort and When Cooperating with Slackers

Paul Ibbotson[1]* Christoph Hauert[2] and Richard Walker[3]

*corresponding author
[1, 3] Open University,
Walton Hall,
Milton Keynes.
MK7 6AA
England.
(+44) 1908 858462
paulibbotson@open.ac.uk

[2]Department of Mathematics
The University of British Columbia
1984 Mathematics Road
Vancouver, B.C.
Canada, V6T 1Z2

Supplementary Information.

Each participant plays a set of five games lasting 10 seconds per game. The ball advanced the same unit of distance per click. Seven clicks moved the ball from its starting position to the edge of the ramp while the 8th click caused it to fall in the bucket. Each game involves the participant and a virtual player whose activity is simulated by the software. The click activity of the real player is recorded as a moving average of five clicks recalculated every 50 milliseconds. The moving average is used as the basis for the click frequency of the virtual player. The virtual player's click activity is random, but with a weighting factor that seeks to generate a click frequency whose ratio to that of the real player is one of the weights 1/2, 2/3, 1, 3/2, 2.

In each set of five games each of these weights is used exactly once, in a randomly determined order. The real player is not directly aware of the existence of these weights and the only information they have about the virtual player is what they can observe from its actions. At the end of each game the total number of clicks made by each player, the virtual one and the real one, are sent to the web server and recorded there. The actual ratio for a particular game approximates the assigned weight, but the match is unlikely to be exact because a real player will often vary their effort during the course of the game, and the response of the virtual player is the outcome of a (pseudo) random process.
